# Proteomic Analysis of S-Acylated Proteins in Human B Cells Reveals Palmitoylation of the Immune Regulators CD20 and CD23

**DOI:** 10.1371/journal.pone.0037187

**Published:** 2012-05-17

**Authors:** Corinne Ivaldi, Brent R. Martin, Sylvie Kieffer-Jaquinod, Agnès Chapel, Thierry Levade, Jérôme Garin, Agnès Journet

**Affiliations:** 1 Laboratoire de Biologie à Grande Echelle, IRTSV, CEA, Grenoble, France; 2 INSERM, U1038, Grenoble, France; 3 Université Joseph Fourier, Grenoble, France; 4 Department of Chemistry, University of Michigan, Ann Arbor, Michigan, United States of America; 5 INSERM, UMR1037, Toulouse, France; 6 Centre de Recherche en Cancérologie de Toulouse, Université Toulouse III Paul-Sabatier, Toulouse, France; 7 Laboratoire de Biochimie Métabolique, Institut Fédératif de Biologie, Hôpital Purpan, Toulouse, France; University Paris Sud, France

## Abstract

S-palmitoylation is a reversible post-translational modification important for controlling the membrane targeting and function of numerous membrane proteins with diverse roles in signalling, scaffolding, and trafficking. We sought to identify novel palmitoylated proteins in B lymphocytes using acyl-biotin exchange chemistry, coupled with differential analysis by liquid-chromatography tandem mass spectrometry. In total, we identified 57 novel palmitoylated protein candidates from human EBV-transformed lymphoid cells. Two of them, namely CD20 and CD23 (low affinity immunoglobulin epsilon Fc receptor), are immune regulators that are effective/potential therapeutic targets for haematological malignancies, autoimmune diseases and allergic disorders. Palmitoylation of CD20 and CD23 was confirmed by heterologous expression of alanine mutants coupled with bioorthogonal metabolic labeling. This study demonstrates a new subset of palmitoylated proteins in B cells, illustrating the ubiquitous role of protein palmitoylation in immune regulation.

## Introduction

Protein S-acylation (or S-palmitoylation, hereafter referred to as palmitoylation) is a common post-translational modification covalently linking long-chain fatty acids (especially 16-carbon palmitic acid) to cysteine thiols in proteins via a labile thioester bond, which can be highly dynamic [1]. Protein acyltransferases and protein acylthioesterases regulate enzymatic cycling of palmitoylation of many proteins [2], [3], providing a distinct pathway for precise regulation of membrane association. Palmitoylation facilitates membrane association of numerous soluble proteins, yet many palmitoylated proteins like ion channels, cell adhesion molecules, G-protein coupled receptors, and immune receptors are integral membrane proteins. For these transmembrane proteins, palmitoylation may act as more than a simple membrane tether. Indeed, palmitoylation has been shown to spatiotemporally regulate many cellular processes, including targeting to specific subcellular compartments or membrane subdomains, trafficking, protein-protein interactions, protein function and protein stability (reviewed in [2], [4]–[6]). For many proteins, palmitoylation mediates lipid raft association [7], which describes membrane microdomains enriched in cholesterol, glycosphingolipids and signaling proteins [8]. Recently, Freeman and coworkers [9] provided proteomic evidence suggesting lipid rafts and tetraspanin-rich microdomains are enriched in specific palmitoylated proteins.

The recent development of palmitoyl-protein enrichment methods has significantly advanced the study of palmitoylated proteins. Acyl-biotin exchange (ABE) [10] is based on hydroxylamine selective cleavage of thioester bonds, followed by free thiol capture via a biotinylated thiol reagent. Biotinylated proteins are then purified by affinity on immobilized streptavidin. Using this approach, Roth *et al.* identified and validated 35 proteins out of 58 novel candidate yeast palmitoylated proteins [11]. Kang *et al.* extended this method to study whole rat brain, embryonic rat neurons or rat synaptosomes, confirming 21 novel palmitoylated proteins out of >200 candidates [12]. Others have recently reported similar studies in african trypanosomes [13], human platelets [14], and macrophages [15]. This approach was recently optimized to eliminate several manipulations by capturing hydroxylamine-sensitive cysteines using a thiol-reactive resin [16].

A second method utilizes the metabolic incorporation of a bioorthogonal palmitate analog into endogenous sites of palmitoylation, which may be subsequently conjugated *in vitro* with a tag such as biotin [17]–[20]. Recently, we used this technique to purify palmitoylated proteins from Jurkat T cells, identifying 125 high confidence and about 200 medium confidence potentially palmitoylated proteins [19]. Other studies by Hang's group used a similar approach to identify palmitoylated proteins in dendritic cells, identifying IFITM3 as an important palmitoylated protein involved in viral infection [21].

Given the diversity of palmitoylated proteins identified in each of these experiments, and the growing understanding of protein palmitoylation in immune regulation, we sought to identify novel palmitoylated proteins in B lymphocytes. Palmitoylated proteins from immortalized B lymphoid cells were enriched using ABE chemistry, and samples prepared with or without hydroxylamine treatment were compared by a semi-quantitative differential proteomic analysis based on spectral counting [22]. This analysis method sums the number of tandem mass (MS2) spectra for a protein, which has been demonstrated to be correlated with the relative protein abundance, and is widely accepted for the relative quantification of proteins between samples [23]–[25]. This strategy led to the identification of 53 previously confirmed palmitoylated proteins and 95 candidate palmitoylated proteins, including numerous major histocompatibility complex (MHC) proteins. Among these candidates, we focused on the B-lymphocyte antigen CD20 and the low affinity immunoglobulin epsilon Fc receptor (CD23). These two surface proteins are important immune regulators with effective and/or potential therapeutic benefits when used as markers to deplete B lymphocytes in B cell lymphoproliferative disorders, such as follicular lymphoma or chronic lymphocytic leukemia, autoimmune diseases such as rheumatoid arthritis, or allergic disorders such as allergic asthma [26]–[29]. This study validates CD20 and CD23 as novel, B cell palmitoylated proteins.

## Results

**Figure 1:**
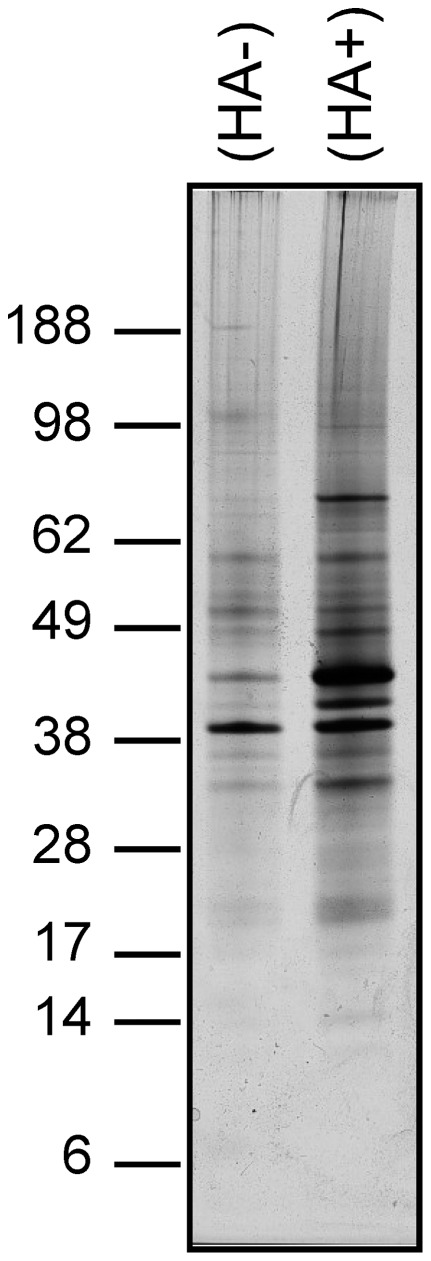
Palmitoylated proteins from human B lymphoid cells are enriched following ABE purification. Proteins from B lymphoid cells membranes were subjected to ABE purification with (HA+) or without (HA−) hydroxylamine treatment, resolved by SDS-PAGE on a 4–12% gradient gel, and detected by silver nitrate staining.

**Figure 2:**
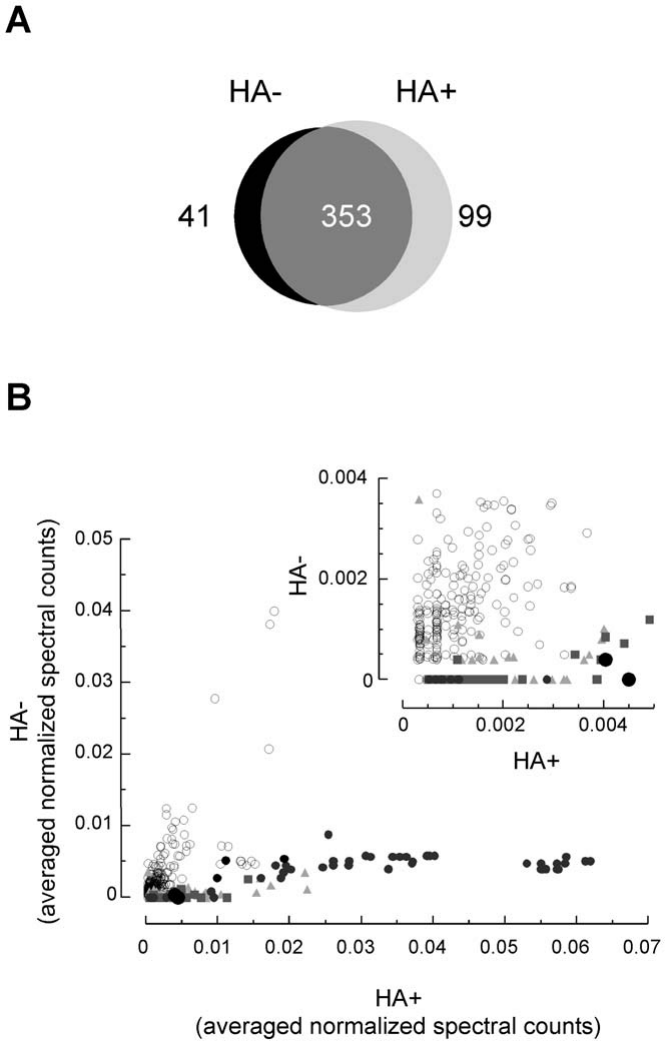
Graphical analysis of the B cell palmitoyl-proteome. (A) Overlap between proteins identified in the HA− or HA+ samples. Results from the triplicate analyses were merged and led to a list of 493 proteins that distribute as depicted in the Venn diagram. The numbers of proteins identified exclusively in the HA− samples, common to HA− and HA+ samples or exclusively identified in the HA+ samples are indicated. (B) Proteins identified in the HA+ samples (452 proteins) were plotted by their averaged normalized spectral counts in the HA+ (x axis) and the HA− (y axis) samples. The inset shows an expanded view of the lower values zone. Novel candidate palmitoylated proteins (•) cluster along the x-axis, as do established palmitoylated proteins (▴) and many candidate palmitoylated proteins identified in previous proteomic studies (▪). ○, proteins that were not significantly enriched, according to our statistical analysis; •, CD20 and CD23.

In order to identify potentially novel palmitoylated proteins from human B lymphoid cells, ABE experiments were performed following the protocol described by Drisdel and Green [10]. Thioester-containing proteins were distinguished from non-specifically enriched proteins by comparing samples treated in parallel with (HA+) or without (HA−) hydroxylamine (Figure 1). Three independent pairs of samples were prepared and analyzed by mass spectrometry. Results from all samples were merged, and this led to the identification of 493 non-redundant proteins identified by at least 2 unique peptides, 452 of which were present in the specific HA+ sample (Figure 2A and Table S1). Fifty-nine of the identified proteins were isoforms of MHC class I or class II proteins, which have in some cases been described as palmitoylated [30]. Fifty-three previously confirmed palmitoylated proteins were identified (Table S1), including the transferrin receptor, the cation-dependent mannose-6-phosphate receptor, flotillins 1 and 2, vesicular trafficking proteins (syntaxins 6, 7, 8 and 12, SNAP23), heterotrimeric G protein alpha subunits, and several members of the small GTPases ras family. All proteins present in the HA+ samples were plotted by their averaged normalized spectral count in HA+ (x-axis) and HA− (y-axis) samples (Figure 2B). As expected, most of the known palmitoylated proteins clustered along the x-axis (grey squares), as did the proteins later identified as palmitoylated candidates (see below; black circles).

### Identification of potentially novel palmitoylated proteins

**Figure 3:**
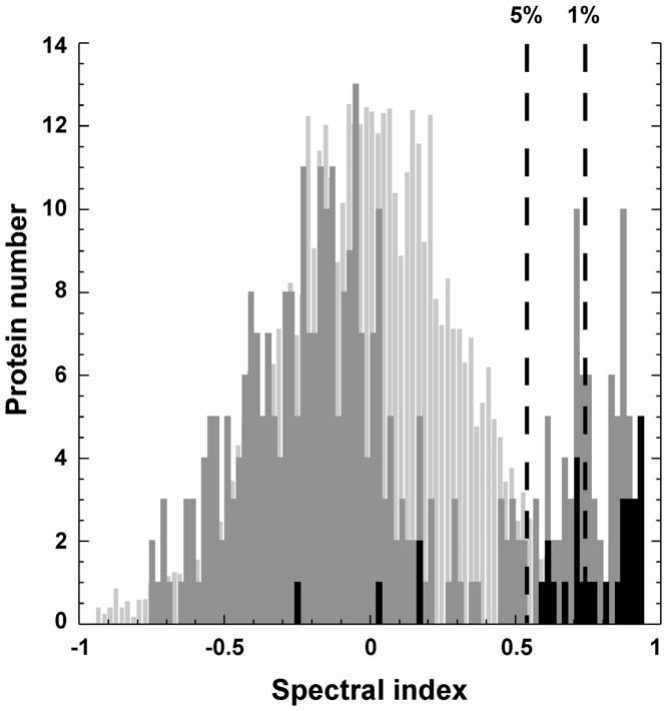
Spectral index distribution of the identified proteins. The distribution of the SpI of the 353 proteins identified in both HA− and HA+ samples is represented as an histogram (medium grey). The random distribution of the SpIs was calculated according to Fu et al. [32], in order to determine the SpI thresholds corresponding to the 5% and 1% confidence intervals (respectively 0.54 and 0.74), and is shown in the background (light grey). The distribution of the known palmitoylated proteins identified in this study is superimposed in black.

Putative palmitoylated proteins were partitioned into two groups: proteins exclusively identified in specific (HA+) samples, and proteins common to both control (HA−) and HA+ samples. Data from the first group were filtered to proteins with at least 5 spectral counts, which led to the identification of 57 proteins. This group includes 16 known palmitoylated proteins, 30 proteins annotated as candidates in previous proteomic studies [9], [12], [14]–[16], [19], [21], [31] and 11 novel potential palmitoylated proteins (Tables S2 and S3). In the second group (353 proteins), candidates were searched by a spectral-count based analysis of their specific enrichment in HA+ samples [22], [24]. This enrichment, indicative of potential palmitoylation, was evaluated using a spectral index (SpI) that takes into account both the relative protein abundance (assessed by normalized spectral counts) and the number of replicates in which the protein has been found [32] (Table S3). As depicted in Figure 3, the SpI distribution of the proteins was clearly bimodal. Statistical analysis showed that proteins displaying a SpI higher than 0.54, representing most proteins from the right peak, were significantly enriched (p<0.05) in the HA+ sample over the HA− sample, and consequently annotated as putative thioester-containing proteins. As expected, the SpI distribution of previously confirmed palmitoylated proteins (Table S3 and Figure 3, black bars) demonstrated a strong tendency to score high SpIs (close to +1). Twenty-eight out of the 32 known palmitoylated proteins identified in both HA− and HA+ in this study displayed a SpI higher than 0.54, and 18 of them had an SpI higher than 0.73 (1% significance level threshold value), illustrating the effective enrichment of palmitoylated proteins in the HA+ samples. Using this approach, 54 additional candidate palmitoylated proteins, comprising 44 MHC proteins, 8 proteins identified in previous proteomic studies [9], [12], [14]–[16], [19], [21], [31] and 2 novel candidates, were annotated (Tables S2 and S3). Among these candidates, the protein that displays the lower spectral count is the B-lymphocyte antigen CD20, with 38 spectra identified in HA+ and one in HA−.

**Figure 4:**
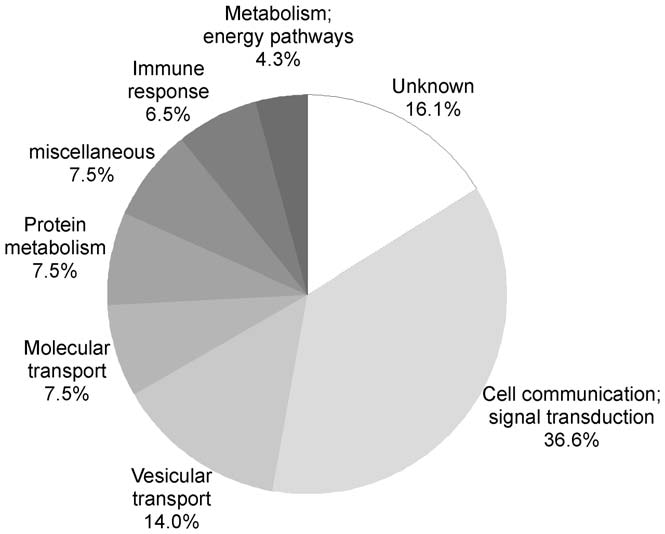
Functional distribution of the candidate palmitoylated proteins. The 93 proteins meeting the determined criteria for being candidates (including known palmitoylated proteins, but depleted from MHC molecules) were classified according to the biological processes they are associated with, as described in the Human Protein Reference Database. The “transport” category has been further manually subdivided in “molecular transport” and “vesicular transport” categories.

Altogether, we identified 95 putative palmitoylated proteins, out of which 46 are MHC molecules and 38 have been annotated as candidates in previous palmitoyl-proteome studies [9], [12], [14]–[16], [19], [21], [31] (Tables S2 and S3). The remaining 11 proteins are completely novel candidates. All proteins meeting our criteria for being considered as potentially palmitoylated, except for MHC molecules, were classified by biological process, as provided by the Human Protein Reference Database [33]. Diverse processes throughout the cell are represented, including cellular signaling (36.6%), vesicle fusion and membrane trafficking (14%), and immune response (6.5%; Figure 4). Among the novel candidates, three are annotated as implicated in signal transduction (the interferon induced transmembrane protein 1 IFITM1, the tumor necrosis factor receptor superfamily member 8 TNFRSF8 or CD30, and the B-lymphocyte antigen CD20) and two in the immune response (CD74 and the low affinity immunoglobulin epsilon Fc receptor FCER2 or CD23).

### The B lymphocyte specific immune regulators CD20 and CD23 are novel palmitoylated candidates

The main goal of this study was to identify B cell novel palmitoylated proteins. Among the proteins specifically enriched in the HA+ samples, we focused on two immune regulators, CD20 and CD23. CD20 was identified by 5 peptides (39 MS2 spectra), corresponding to 4 different sequences covering 11.3% of the protein sequence (Table 1). CD20 was exclusively found in the HA+ sample in two out of three experiments, displaying a SpI of 0.88 that was included in the 1% confidence interval. Similarly, CD23 was exclusively identified in the HA+ samples for the three replicates, by a total of 10 peptides (43 MS2 spectra), corresponding to 8 unique sequences covering 32.3% of the protein sequence (Table 1).

**Table 1:** List of the peptides allowing the identification of CD20 and CD23.

protein	peptides	position	length
CD20	MESLNFIR (oxidized form)	149–156	8
	MESLNFIR (dioxidized form)	149–156	8
	SNIVLLSAEEK	225–235	11
	SNIVLLSAEEKK	225–236	12
	EEVVGLTETSSQPK	244–257	14
CD23	MEEGQYSEIEELPR	1–14	14
	MEEGQYSEIEELPR (oxidized form)	1–14	14
	SQSTQISQELEELR	83–96	14
	SQSTQISQELEELRAEQQR	83–101	19
	SQELNERNEASDLLER	125–140	16
	NEASDLLER	132–140	9
	YACDDMEGQLVSIHSPEEQDFLTK	189–212	24
	GEFIWVDGSHVDYSNWAPGEPTSR	230–253	24
	SQGEDCVMMR	254–263	10
	SQGEDCVMMR (oxidized form)	254–263	10

### Validation of CD20 and CD23 as novel palmitoylated proteins

**Figure 5:**
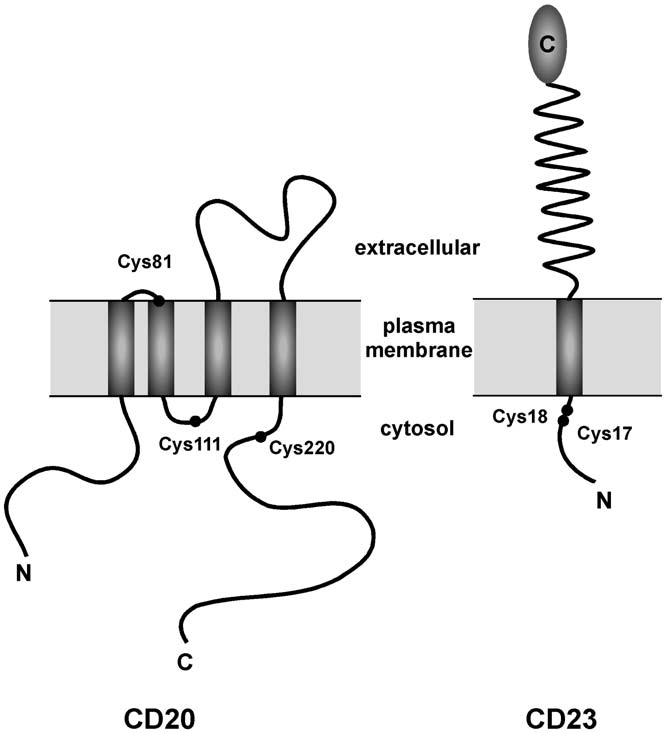
Schematic representation of CD20 and CD23. Cysteines that are potential sites of palmitoylation are indicated, as well as N and C termini. For the sake of simplicity, CD23 is depicted as a monomer, although CD23 molecules are assembled in trimers in the membrane [36]. The CD20 scheme is adapted with permission from Ernst *et al*. [35]. Copyright (2005) American Chemical Society.

CD20 is a non-glycosylated four-pass transmembrane protein with a large extracellular loop located between the third and fourth transmembrane segments [34] (Figure 5). Two out of five cysteines are located on the extracellular loop, excluding them as potential palmitoylation sites. The three intracellular cysteines (Cys^81^, Cys^111^ and Cys^220^) are candidate sites of CD20 palmitoylation. Cys^81^ is predicted to be embedded within the first or the second transmembrane domain, Cys^111^ reside between the second and the third transmembrane domains in the cytoplasmic loop and Cys^220^ is in the C-terminal intracellular tail [35].

CD23 is a single-pass type II integral membrane protein (Figure 5) which is present as a homotrimer in the cell membrane [36]. The short N-terminal cytoplasmic tail contains two cysteines (Cys^17^ and Cys^18^), which are likely sites of palmitoylation. These cysteines are juxtaposed to the predicted cytoplasmic/transmembrane domain boundary and surrounded by clusters of hydrophobic and positively charged amino-acids, characteristics that are often found in transmembrane palmitoylated proteins [37].

In order to validate the palmitoylation of CD20 and CD23, we developed a heterologous expression system, where palmitoylation of these specific candidates was assessed by transient transfection of plasmids encoding CD20 and CD23 tagged with a C-terminal (His)_6_ epitope. Additionally, in order to identify the palmitoylated cysteines in CD20 and CD23, we generated single point mutations in CD20 (C81A, C111A, C220A) and CD23 (C17A and C18A). As aberrant palmitoylation frequently occurs after mutating a cysteine in a context of neighbouring cysteines [5], [38], the corresponding CD20 and CD23 double mutations (C111A/C220A for CD20 and C17A/C18A for CD23) were also analyzed. The predicted topology of CD20 led us to build only the C111A/C220A CD20 double mutant [35] (Figure 5).

**Figure 6:**
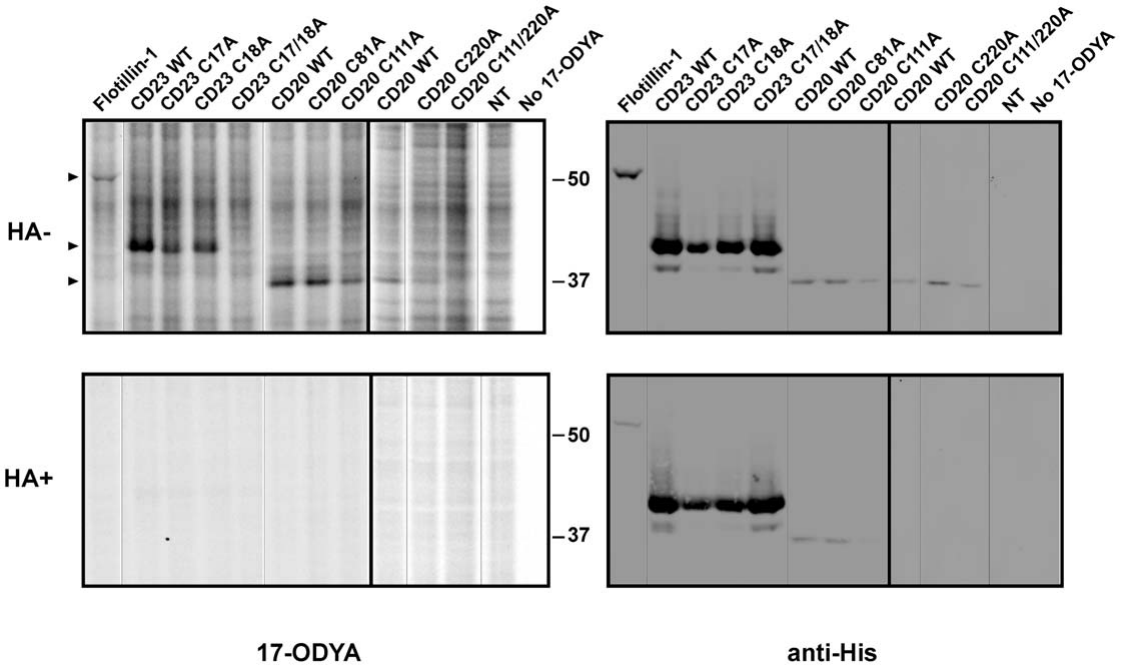
The immune regulators CD20 and CD23 are novel palmitoylated proteins. HEK293T cells were transfected with plasmids encoding His-tagged versions of flotillin-1, CD20, CD23 and Cys-to-Ala mutants of CD20 and CD23, and metabolically labeled with 17-ODYA. Cell membranes were then subjected to click chemistry prior to separation of proteins by SDS-PAGE on a 10% acrylamide gel. Proteins were treated (lower panels) or not (upper panels) with hydroxylamine before gel loading. Left panels: Scans of the fluorescent proteins. Right panels: Immunodetection of the exogenous proteins with an anti-His antibody. Arrowheads indicate the position of overexpressed proteins. The figure is a composite of two gels, as shown by a thick line. Lanes separated by a thin line were not originally contiguous in the gel, but were placed next to each other in the figure for the sake of clarity. Gels and blots were cropped around the region of interest.

In order to unambiguously annotate CD20 and CD23 as novel palmitoylated proteins, transfected HEK-293T cells were metabolically labeled with 17-ODYA for palmitoylation analysis (Figure 6). This orthogonal labeling approach uses metabolic labeling to attach a fatty acid analogue in native cells, providing an additional validation of protein palmitoylation. Flotillin-1, a well-established palmitoylated protein [39], was used as a positive control. After labeling with 17-ODYA for 6 hours, cell membranes were mixed with rhodamine-azide, TCEP, TBTA, and copper sulphate under standard click chemistry conditions. Samples were then separated by SDS-PAGE and analyzed using a fluorescent scanner to detect 17-ODYA labeled proteins. For each transfected protein, a hydroxylamine-sensitive fluorescent band appeared in the transfected samples, indicative of protein palmitoylation (Figure 6, left panels). The identity of the fluorescent species was finally checked by western blot using an anti-His antibody, demonstrating alignment between unique fluorescent 17-ODYA labeled bands and the (His)_6_-tagged species (Figure 6, upper right panel). For all three proteins, one band only was detected at the expected size for the complete tagged protein, *i.e.* 51 kDa for flotillin-1, 37 kDa for CD20 and 43 kDa for CD23. Based on these results, we conclude that CD20 and CD23, like flotillin-1, are valid palmitoylated proteins measured by two distinct palmitoylation detection schemes.

Mutational analysis also demonstrated that the C81A and C111A mutants of CD20, but not the C220A mutant, incorporated equivalent amounts of 17-ODYA, relative to their expression levels. The double mutant C111A/C220A completely eliminated 17-ODYA incorporation. Accordingly, CD20 is palmitoylated on its intracytoplasmic cysteines Cys^111^ and/or Cys^220^. Single mutants (C17A, C18A) of CD23 did not reduce the incorporation of 17-ODYA, but the double mutant (C17A/C18A) completely abrogated the observed labeling. Therefore, CD23 is palmitoylated on either Cys^17^ and/or Cys^18^.

## Discussion

In order to identify novel palmitoylated proteins in human B lymphocytes, we used the ABE method [10] followed by a semi-quantitative mass spectrometry analysis. The analysis of three independent purifications allowed us to identify 493 proteins from human B lymphoid cells, among which 53 were known palmitoylated proteins. These included flotillins-1 and -2, vesicular trafficking proteins, Ras-family GTPases, G-α subunits, kinases and receptors, similarly to previous proteomic studies [9], [12], [14], [15], [31]. Candidate palmitoylated proteins were identified by semi-quantitative analysis using spectral counting approaches [22] and comparison between samples prepared with or without hydroxylamine treatment. The calculation of a spectral index [32] led to the identification of 95 candidate palmitoylated proteins.

Given the overlap with previous efforts to profile palmitoylated proteins in immune cells, we focused our interest on two proteins involved in the immune response, CD20 and CD23, which were both identified unambiguously as candidate palmitoylated proteins in the present study. Interestingly, CD20 and CD23 are both effective or promising targets for immunotherapy of haematological malignancies, autoimmune diseases or allergy disorders [26]–[29]. CD20 expression is restricted to pre-B and mature B cells and regulates B cell activation and proliferation [40]. CD23, also known as the low affinity receptor for immunoglobulin E, is a transmembrane receptor expressed by various haematopoietic and epithelial cells that participates in tuning the immune response by regulating IgE production [41], [42].

CD20 and CD23 palmitoylation was validated by heterologous expression and metabolic labeling with the palmitate analog 17-ODYA. CD20 and CD23 palmitoylation sites were also assessed by systematic mutational analysis of intracytoplasmic or membrane-embedded cysteines [5], [37]. Although the single mutations had no or only a limited effect on the protein palmitoylation, both double mutations C111A/C220A (in CD20) and C17A/C18A (in CD23) completely prevented this modification. This implies that the palmitoylation site in CD20 or in CD23 actually resides in the pairs of cysteines tested. It has been previously observed that single mutants in neighbouring cysteines could be palmitoylated because of aberrant modification of the remaining cysteines [5], [38]. CD23 is clearly in this configuration of vicinal cysteines. Concerning CD20, Cys^111^ belongs to the intracellular loop between the second and the third transmembrane domains, while Cys^220^ is located in the C-terminal tail. Despite their distance in the primary sequence, they could nevertheless be in close proximity in the folded protein and behave like vicinal cysteines in these palmitoylation experiments. Our observation that the C220A mutation was more effective in reducing CD20 palmitoylation than did the C111A one, indicative of Cys^220^ modification, was corroborated by previously published data. Indeed, the presence of post-translational modifications in the C-terminal region, such as phosphorylation or acylation, had been proposed, as the observed molecular weight of a CD20 C-terminal fragment resulting from protease digestion of intact cells was found to significantly exceed its expected size (21 kDa instead of 14; [34]). Besides, Cys^111^ is the only CD20 cysteine that is unique to humans and not conserved in the mouse CD20 sequence [35], suggesting that palmitoylation of this residue is unlikely to be essential for CD20 function.

Protein palmitoylation is a key regulatory mechanism for numerous cellular processes that include membrane targeting, segregation to membrane microdomains (*i.e.* lipid rafts), protein stability, protein-protein interactions or cellular signaling [2], [4]–[6]. Because CD20 and CD23 are both associated to cellular signaling cascades and display multiple interactions with immune actors such as the B cell receptor or the complement receptor CD21 [41], [43]–[46], their respective palmitoylation might be one of the switches involved in the tuning of CD20 or CD23 immune activity and in the cell response to anti-CD20 or anti-CD23 immunotherapy.

In conclusion, our analysis of the B lymphocyte palmitoyl-proteome led to the selection and validation of the two immune regulators CD20 and CD23 as B cell novel palmitoylated proteins. These results open the way to future studies aiming at deciphering the role of CD20 and CD23 palmitoylation in immune activation and in cellular responses to B cell directed immuno-, radioimuno- or chemoimmunotherapies.

## Materials and Methods

### Chemicals and reagents

Except when mentioned, chemicals were from Sigma-Aldrich. Latrunculin B was purchased from Calbiochem. Complete, EDTA-free, protease inhibitor cocktail and the Fugene HD transfection reagent were from Roche. Hydroxylamine was obtained from Alfa Aesar. EZ-Link biotin-HPDP was from Thermo Scientific. 17-octadecynoic acid (17-ODYA) was obtained from Cayman. The Colloidal Blue solution (Biosafe) and the BCA assay were from BioRad. The modified trypsin (sequencing grade) was purchased from Promega. Formic acid was obtained from Aristar.

RPMI-GlutamaxI, penicillin-steptomycin-glutamine, and Dynabeads M-280 streptavidin were obtained from Invitrogen. FBS was from Invitrogen or Gemini. DMEM (High Glucose) and D-PBS were purchased from Mediatech.

### Palmitoylated protein purification from B lymphoblasts

A human Epstein-Barr virus-transformed B lymphoid cell line was established in the lab from a donor whose parents had given written informed consent for the establishment and storage of the cell line, according to the protocols approved by the ethics review board of the Toulouse Hospital. Research was performed in compliance with the Declaration of Helsinki. Cells were grown in RPMI-GlutaMaxI containing 10% FBS.

Palmitoylated proteins were purified from 200×10^6^ B cells according to Wan *et al.* [47], except that Tris buffer was replaced by Hepes. As preliminary experiments had demonstrated the very high abundance of actin in our samples, that hampered identification of other proteins, we also added a cell incubation step in the presence of 400 µM latrunculin B in complete culture medium for 30 minutes at 37°C prior to harvesting, in order to deplete the sample from polymerized actin [48]. Cells were then pelleted by centrifugation, resuspended at 50×10^6^ cells/mL (in 50 mM Hepes, pH 7.4, 150 mM NaCl, 5 mM EDTA, pH 8.0, 0.1% Triton X-100, protease inhibitors), and finally broken by 4 cycles of freezing/thawing before sonication. Total membranes were recovered by high-speed centrifugation (100 000×g, 1 h, 4°C) and resolubilized in 50 mM Hepes, pH 7.4, 150 mM NaCl, 5 mM EDTA, pH 8.0, 1.7% Triton X-100, 10 mM N-ethylmaleimide (NEM), protease inhibitors. Then, proteins were extracted by methanol-chloroform precipitation. Resolubilized proteins were incubated successively with NEM for blockade of free thiols, and with hydroxylamine (HA+ sample) to release the thioester-linked palmitate and restore the modified cysteines to thiols. Formerly thioester-containing (mainly palmitoylated) proteins were biotinylated using a thiol-reactive biotinylation reagent (EZ-Link biotin-HPDP) and finally captured on streptavidin-coated magnetic beads (Dynabeads M-280 streptavidin). Specifically bound proteins were eluted by β-mercaptoethanol reduction of the protein-biotin disulfide linkage. An essential control of non-specific biotinylation and binding to the streptavidin matrix consisted in replacing hydroxylamine by 50 mM Hepes, pH 7.4, in half of the NEM-treated sample (HA− sample).

### Palmitoylation assays

HEK293T cells were cultured in DMEM (High Glucose) supplemented with 10% FBS and 1% penicillin-steptomycin-glutamine. Cells were transiently transfected using purified plasmid DNA and Fugene HD transfection reagent. Twelve hours after transfection, cells were labeled with 20 µM 17-ODYA for 8 hours, washed twice with D-PBS, and then harvested and frozen at −80°C. Cell pellets were later sonicated in D-PBS and separated into soluble and insoluble fractions by ultracentrifugation for 45 minutes at 100 000× g. The insoluble pellet was resuspended by sonication in D-PBS, and then quantified using the BCA protein assay using a microplate reader. Cell lysates were normalized to 1 mg/mL. To 100 µL of each lysate, click chemistry reagents were added at room temperature to yield a final concentration of 20 µM rhodamine-azide, 1 mM Tris(2-carboxyethyl)phosphine (TCEP), 100 µM Tris[(1-benzyl-1H-1,2,3-triazol-4-yl)methyl]amine (TBTA), and 1 mM CuSO4 in PBS. After 1 hour, half of the sample (50 µL) was transferred to a fresh tube and mixed with 1 M hydroxylamine and boiled for 5 minutes. All samples were mixed with 4× SDS sample loading buffer and 2/3 (33 µg) of the sample was loaded on a 10% SDS-PAGE gel, separated over 850 volt-hours, and analyzed using a Hitachi FMBIO-II flatbed fluorescence scanner. Gels were then transferred to nitrocellulose (100 volt-hours), blocked with 5% non-fat dry milk in Tris-buffered saline Tween-20 buffer (TBST), incubated overnight in 2% bovine serum albumin with 1∶2000 mouse anti-His (C-term) (Invitrogen) in TBST, washed, and incubated with 1∶10 000 goat anti-mouse IRDye 800CW (LiCOR) for 1 hour. After washing, the membrane was imaged using an Odyssey Imager (LiCOR).

### SDS-PAGE analyses

ABE purified proteins were separated on SDS-polyacrylamide gels (4–12%, NuPage, Invitrogen). They were revealed either by silver staining for analytical purpose or by colloidal blue staining for mass spectrometry analysis.

### Cloning and DNA manipulation

cDNAs encoding CD23a and flotillin-1 tagged with a C-terminal V5-His_(6)_ sequence were purchased as pDEST51-based plasmids (respectively OCAAo5051D0795 and OCAAo5051H0577, Imagenes, Source BioScience LifeScience). The CD20 cDNA was amplified by PCR from human B cells cDNA, with the following primers: 5′-ATTGGATCCGCCGCCATGACAACACCCAGAAATTC-3′ and 5′-GCGCTCGAGAGGAGAGCTGTCATTTTC-3′, and cloned into pcDNA3.1/myc-His (Invitrogen) at the *Bam*HI and *Xho*I sites to generate pcDNA3.1-CD20-myc-His.

Cys-to-Ala point mutations were generated by site-directed mutagenesis (QuickChange Lightning Site-Directed Mutagenesis kit, Agilent Technologies), according to the manufacturer instructions. The CD20 insert was first transferred into the pUC18 vector for directed mutagenesis, and then the mutated insert was returned back to the pcDNA3.1/myc-His vector, using *Nde*I and *Xba*I sites at both steps. The CD23 insert was transferred by use of the Gateway technology (Invitrogen) into a pENTR221 vector, subjected to directed mutagenesis and returned to the pDEST51 Gateway vector. The primers used for generating the mutations were the following: 5′-GGATCTATGCACCCATCGCTGTGACTGTGTGGTACC-3′ and 5′-GGTACCACACAGTCACAGCGATGGGTGCATAGATCC-3′ for CD20 C81A; 5′-CGGAGAAAAACTCCAGGAAGGCTTTGGTCAAAGGAAAAATG-3′ and 5′-CATTTTTCCTTTGACCAAAGCCTTCCTGGAGTTTTTCTCCG-3′ for CD20 C111A; 5′-GAGAATGAATGGAAAAGAACGGCCTCCAGACCCAAATCTAAC-3′ and 5′-GTTAGATTTGGGTCTGGAGGCCGTTCTTTTCCATTCATTCTC-3′ for CD20 C220A; 5′-TCCCAGGAGGCGGGCTTGCAGGCGTGGG-3′ and 5′-CCCACGCCTGCAAGCCCGCCTCCTGGGA-3′ for CD23 C17A; 5′-CCAGGAGGCGGTGTGCCAGGCGTGGGACT-3′ and 5′-AGTCCCACGCCTGGCACACCGCCTCCTGG-3′ for CD23 C18A; 5′-GCTTCCCAGGAGGCGGGCTGCCAGGCGTGGGACT C-3′ and 5′-GAGTCCCACGCCTGGCAGCCCGCCTCCTGGGAAGC-3′ for CD23 C17A/C18A.

All constructs were checked by DNA sequencing (Cogenics).

### Mass spectrometry

#### Protein digestion

Protein bands covering the entire migration zone of the SDS-polyacrylamide gel were systematically and manually excised and washed several times by incubation in 25 mM NH_4_HCO_3_ (solution A) for 15 min followed by 50% (v/v) acetonitrile containing 25 mM NH_4_HCO_3_ (solution B) for 15 min. Gel pieces were dehydrated with 100% acetonitrile and then incubated with 7% H_2_O_2_ for 15 min before being washed again with solutions A and B as previously. 0.15 µg of modified trypsin in solution A was added to the dehydrated gel bands for an overnight incubation at 37°C. Peptides were then extracted from gel pieces in three 15 min sequential extraction steps in 30 µL of 50% acetonitrile, 30 µL of 5% formic acid and finally 30 µL of 100% acetonitrile. The pooled supernatants were finally dried under vacuum.

#### Nano-LC-MS/MS analysis

The dried extracted peptides were resuspended in 4% acetonitrile and 0.5% trifluoroacetic acid and analyzed by online nanoLC-MS/MS (Ultimate 3000 and LTQ-Orbitrap, Thermo Fischer Scientific). The nanoLC method consisted in a 40 min gradient ranging from 5% to 55% acetronitrile in 0.1% formic acid at a flow rate of 300 nL/min. Peptides were sampled on a 300 µm×5 mm PepMap C18 precolumn and separated on a 75 µm×150 mm C18 column (Gemini C18, Phenomenex). MS and MS/MS data were acquired using Xcalibur (Thermo Fischer Scientific) and processed automatically using Mascot Daemon software (version 2.3, Matrix Science).

#### Database searching

Consecutive searches against a homemade polypeptide sequence database were performed for each sample using Mascot 2.3. The search database consisted in the concatenation of the SwissProt-Trembl database (release 2010_07, *Homo sapiens* taxonomy, 92 013 entries), the corresponding reverse sequences and classical contaminants (trypsin from pig, proteins from bovine serum, etc., 258 entries). ESI-TRAP was chosen as the instrument, trypsin as the enzyme and 2 missed cleavages allowed. Precursor and fragment mass error tolerance were set respectively at 10 ppm and 0.6 Da. Peptide modifications allowed during the search were: acetyl (N-ter), dioxidation (M), oxidation (M) and trioxidation (C). Proteins identified with a minimum of 1 peptide and with a score higher than the identity threshold (p-value<0.05) were automatically validated using IRMa [49]. Results were downloaded into an MSI database displaying a false positive rate of 0.90% [50]. This database was then treated by a homemade software (hEIDI) for the compilation, filtering, grouping and comparison of the proteins. The filter rejected proteins identified by one peptide only. Finally, identifications of contaminant sequences (trypsin, keratins and non-human proteins) were removed from the protein list. The MS results are available in the PRIDE database (http://www.ebi.ac.uk/pride) under accession number 20197 [51].

#### Semi-quantitative analysis

For each identified protein, its spectral count value was determined and normalized to the total number of assigned spectra in the considered sample. Normalized values were considered for evaluation of the relative protein abundance in each sample using the label free spectral counting method [22]. A spectral index (SpI) was then determined for each protein as indicated in Fu *et al.* [32] to allow comparison between HA- and HA+ samples and help determination of palmitoylated protein candidates.

## Supporting Information

Table S1 **List of identified proteins.** Identification results from all samples were merged and proteins were grouped to respect the principle of parsimony. Only proteins identified by at least two peptides were retained. Proteins that have been previously established as palmitoylated (“Established”) or identified as candidates in previous proteomic screens (“Proteomics”) are indicated along with the corresponding reference. The list of references is below the table. The spectral index has been calculated as described in Fu et al. (J. Proteome Re., 2008;7(3):845–854). NA (not applicable): a spectral index is calculated only for proteins that are present in both HA+ and HA− samples. For the proteins that are not identified in one category of samples (HA+ or HA−), the averaged normalized spectral count of this sample is considered as equal to 0. Sc: spectral count; nsc: normalized spectral count (spectral count of a protein divided by the total number of assigned spectra in the corresponding sample). Exp1 to 3 correspond to the three biological replicates.
(XLS)

Table S2 **Detail of the distribution of the proteins meeting selection criteria.** HA+ only : proteins identified in HA+ samples exclusively. Common proteins: proteins identified in both HA+ and HA− samples. P-value: p-value related to the spectral index analysis.
(XLS)

Table S3 **Candidate palmitoylated proteins.** Proteins meeting our criteria of potential palmitoylation are listed. They include well-established palmitoylated proteins («Established»), proteins previously identified as candidates in proteomic screens («Proteomics»), and novel candidates («Novel»). They are classified according to their associated biological process as indicated in the Human Protein Reference Database and manually reviewed. NA (not applicable): no SpI was calculated for proteins identified exclusively in HA+ samples.
(XLS)
